# Men-Midwives of the past
*A Paper read before the Bristol Medico-Chirurgical Society on 13th March, 1935.


**Published:** 1935

**Authors:** Miles H. Phillips

**Affiliations:** Professor of Obstetrics in the University of Sheffield; Surgeon to the Jessop Hospital, Sheffield


The Bristol
Medico-Chirurgical Journal

"*Scire est nescire, nisi id me
Scire alius sciret.*"

SUMMER, 1935.

### MEN - MIDWIVES OF THE PAST.*

BY

MILES H. PHILLIPS, M.D., F.R.C.S., F.C.O.G.,

*Professor of Obstetrics in the University of Sheffield ;
Surgeon to the Jessop Hospital, Sheffield.*

It is probable that even in remote times some aid was given to the child-bearing woman. Women who had themselves borne children and were thus experienced assisted their neighbours. As civilization advanced some of these women assisted regularly and ultimately as a means of livelihood. Thus arose the midwife.

Whilst the care of the wounded soldier or hunter gradually passed into the hands of the physician, the barber, the surgeon, that of the woman in labour not unnaturally remained with the midwives. Assistance

* A Paper read before the Bristol Medico-Chirurgical Society on 13th March, 1935.

at child-birth was deemed a woman's work, and because of the poor type of woman who plied the trade progress was impossible.

The aid of the priest or man possessed of mystical power, and later of the physician, was called in difficult cases only when the efforts of the midwife were ineffective. It was only at the peak of ancient civilizations, and quite late in our own, that the surgeon personally took charge of labour in occasional cases. In the Old Testament midwives are referred to in several places. Egyptian priests having a certain knowledge of anatomy and medicine overcame some of the difficulties of abnormal labour. Even podalic version was practised.

Later, in Grecian civilization, medical care of the sick made a great advance. Temples of healing were founded, the prototype of our hospitals. But neither women in labour nor sick people who were likely to die were admitted for many generations. It was not until about A.D. 150 that the Emperor Antoninus Pius caused to be built special temples for confinement cases as well as for the dying.

At the time of Hippocrates (400 B.C.) the Grecian midwives were a well-organized group with clearly-defined duties. Their methods were regulated by the physician, and in difficult cases he was called in to assist. However, some contempt was shown him: for this active participation in child-birth he was called a "he-grandmother."

After the destruction of Corinth (B.C. 146) Greek culture migrated to Rome, and Greek physicians were appointed to the Court. Greek medicine progressed at Rome, and culminated in a sound practice of midwifery described in the writings of Soranus, in the second century (98 to 138) after

Christ. Then was set up a standard beyond which there was no further advance for fourteen hundred years—indeed, in many particulars retrogression in midwifery practice took place. Soranus taught rational care and assistance of the women in labour, based on knowledge and not on superstition. He reintroduced podalic version for cases (such as shoulder presentation) previously dealt with by instruments or destruction. He and other men practised midwifery, a custom that two centuries later disappeared from Western civilization for over twelve hundred years. During that time midwifery was not only ignored by the physician but actually prohibited by custom and law. The exclusion of men from the study of child-birth rose to fanatical heights. A Dr. Wertt of Hamburg in 1552 put on the dress of a woman to attend and study a case of labour. He was detected and punished by being burnt to death.

Thus in the Middle Ages the woman was deprived of the aid, however poor it might have been, of the physician, and at the same time urban civilization was making child-birth more hazardous in various ways. Rickets appeared about the middle of the sixteenth century, in England first, then it appeared in Germany and all the northern parts of Europe. The difficulty produced by the consequent deformity of the pelvis was probably one of the reasons which led to the physician or surgeon being again called in to assist. Indeed, it was not until the sixteenth century that progress in the art of midwifery recommenced in Europe.

In that century the celebrated French surgeon, Ambroise Paré, advocated that the operation of podalic version should be used in those cases where the child was not in the normal position. This manœuvre was the chief factor in bringing about the emancipation of the child-bearing woman from the exclusive hands of the ignorant midwives who had been in control since the fall of the Roman Empire. It actually laid the foundation of the modern art of obstetrics. The physician could now assist the woman in difficult labour without mutilating the child.

In Paré's time a school of midwives was founded in Paris (the Hôtel Dieu). The women who graduated there were vastly superior to the ignorant old hags who had trundled their obstetrical chairs from house to house. The customs of the time changed; trained and supervised midwives were a great advance; they were better able to recognize difficulties and more ready to call in the assistance of the surgeons who had been partly responsible for their training.

At first men were called in only when the midwife found herself in difficulty, but before long their aid was sought in normal cases. Thus Louis XIV. employed men to attend two of his mistresses. Some writers hint that this was done because secrecy was desired, but it may well be that the king had come to consider the presence of a man to be an extra safeguard. Monsieur Boucher attended Mademoiselle La Vallière, and in 1670 Monsieur Jules Clément confined Madame de Montespan. The king, it is said, watched the procedure from the shelter of a curtain, and was very pleased with the decorous conduct of the men-wives. Clément was honoured with the new and more dignified title of accoucheur.

The custom of employing a man soon spread amongst the ladies of the Court, and the king's own heir was ushered into the world by Monsieur Clément in 1682.

The knowledge of normal and abnormal labour thus gained by these French accoucheurs soon led to the school of midwifery in Paris gaining such a great reputation that doctors from all over Europe studied there. Text-books were written by both French midwives and doctors. These were brought to England, and before long translations were published in London. They gradually replaced the hand-books for midwives which were in use by the English ignorant Gamp. These English books contain little else than superstitious twaddle. The author of one of them is the hero of that charming story "A Doctor of Medicine" in Rudyard Kipling's *Rewards and Fairies.* This was a certain Nicholas Culpeper who styled himself "Gent. Student in Physick and Astrology" and wrote *A Directory for Midwives.* I own an edition published in 1675 which is described on the title-page as being "Newly corrected from many gross Errors," so it is not the first edition.

Culpeper evidently knew nothing of practical midwifery, and if there is any useful advice in the book it is hopelessly hidden in a mire of herbalistic and astrological quackery. This is sad, for Culpeper was a kindly soul, and wished to be helpful to the community and to midwives in particular. He dedicates the book thus:—

"To the Midwives of England, Nicholas Culpeper wisheth success in their Office in this World, and a Crown of Glory in that to come.

"Worthy Matrons:

"You are of the Number of those whom my Soul loveth, and of whom I make dayly mention in my Prayers."

Here is a sample of the rubbish he wrote. To facilitate delivery in cases of dry labour he gives no less than sixteen prescriptions: "The juyce of Leeks being drunk with warm water, hath a mighty operation to cause speedy delivery ;" or, "Give a woman in such a case another Woman's Milk to drink, it will cause speedy delivery, and almost without pain ;" and, "Take a Swallow's nest, and dissolve it in water, strain it, and drink it warm, it gives delivery with great speed, and much ease."

Apparently fearing that these results may not be good enough for some midwives, he states that "The stone Ætitis held to the Privities, instantly draws away both child and afterburden ; yea, draws out womb and all, if you remove it not instantly after they are come away, its Magnetick vertue is such. If you do any mischief that way, the fault is not mine, you are fore-warned of it." I am afraid that such a statement stamps him as an insincere humbug. Unfortunately his and similar writings were all that were available for our Sairey Gamps for many years. Even in 1752 William Smellie, the greatest of all British obstetricians, writes of Culpeper that "his performances were for many years in great vogue with the midwives, and are still read by the lower sort whose heads are weak enough to admit such ridiculous notions."

It is very regrettable that physicians of the time, with no practical experience of midwifery—though this was not their own fault—should have thought it desirable to give advice, by written word, on a subject so essentially practical.

I have recently come across another instance of this bumptious interference. The book was published in London in 1652. Its title is *The Childbearers Cabinet*, and it is described as "A Short Commentarie, Concerning the Care ought to be had of Women which are with Child." The preface is signed by the initials

A.M. only, and its states that "Since many sad and incommodious things are wont to happen to women with child, and in bringing them into the world by ignorance and carelessness: I thought I should undertake a thing not unbecoming a Christian Physitian."

He is no more helpful than Culpeper, but far less resourceful, having only one suggestion to offer to assist a difficult labour: "Therefore, when there appeareth difficulty in bringing forth the child, Jesus Christ, the onely preserver and saver in danger, is heartily to be called upon, that with his gratious favour he would be pleased to be assistant to the wretched party in travell."

On the other hand, when the woman had been happily delivered he is very busy prescribing various elaborate fomentations, ointments and other applications to the woman's abdomen and "natural parts" during the lying-in period. Finally, he details the ingredients for the cleansing bath which is to be taken from the twentieth day if it be a *male* child, but from the five-and-twentieth day if it be a *female*. A good instance of the fantastic rubbish which is needed to conceal ignorance.

The reluctance of women in labour to be attended by men was based on feelings of modesty. It was overcome by French women long before the English woman became resigned to the assistance of a man. This, of course, applies to cases of natural labour only, for in desperate abnormal labours women had long been willing to accept help at anyone's hands. A striking result was the introduction of the lateral posture in labour, in which the woman's face is turned away from the attendant. This was an English innovation, and was ascribed by Continental writers to *la pudibonderie britannique* (British prudery), and became known as the London posture. Previously, after the birth-chair or stool had been replaced by the bed, she had been placed for delivery on her back, as she is to this day delivered in all other European countries.

References to woman's attitude of mind with regard to the presence of a man are frequent in the writings of the seventeenth and eighteenth centuries. Not a few of them are amusing as well as instructive. A Frenchman, de la Motte, "a sworn surgeon and man-midwife" at Valognes wrote *A General Treatise of Midwifery: Illustrated with upwards of Four Hundred Observations and Reflexions Concerning that Art.* This was translated by Thomas Tomkyns of London, under the supervision of the great William Smellie, and published in London in 1746. The observations were accounts of cases which he had himself attended. No. 63 tells of the disturbing effect on the part of the patient, from fear that Monsieur de la Motte might be able to see too much of her person. He writes :—

"The 28th of July, 1697, the Marchioness of X. . . . whom I was with, was taken in the morning as she awoke with the most violent pains ; I went into her Chamber and finding the child well placed, the waters well formed, and the membranes ready to break at the next pain, I thought it would not be long before she was brought to bed—but I had no sooner laid her on the little bed, but everything changed, by the fear she was in lest I should use my eyes as well as my hands. She could not be got out of this error because she did not mention it, till her waiting maid in whom she put a great confidence, was come to her, to whom she declared the reason of her disturbance ; but being assured that it was even impossible to see her feet, she recovered from her error, the pains returned more violently than before, and she was delivered in a little time, (but) asking her waiting maid whether she was well covered, even in the midst of the strongest pains."

A more remarkable result of this female delicacy is described in a very rare English manuscript, one copy of which is now in the Library of the Royal Society of Medicine and another in the British Museum. It was written in the seventeenth century by Percival Willughby, and is divided into two parts, with two separate titles, "Observations in Midwifery" and "The Country Midwife's Opusculum or Vademecum Shewing the ways and how to deliver any difficult birth bee it naturall or unnaturall." He practised in Derby from 1631 to 1655, then went to London for the better education of his children.

One daughter, however, was practising as a midwife, as is proved by the episode I wish to retail. It is a good instance of the subterfuges required at the time in most households to conceal the presence of the man-midwife. He had, as a matter of fact, to creep into the lying-in room in order to make a vaginal examination unknown to the lady, who was led to think that it was her midwife who was doing so. Here is the story in his own words :—

"In Middlesex, Anno 1658, my daughter, with my assistance, delivered Sir Tenebs Evanks Lady of a living daughter. All the morning my daughter was much troubled and told mee, that shee feared that the birth would come by the buttock. . . . About seven o'clock that night labour approached. At my daughter's request, unknown to the Lady, I crept into her Chamber upon my hands and knees, and returned and it was not perceived by the Lady. My daughter followed mee ; and, I, being deceived, through haste to go away, said that it was the head but she affirmed the contrary ; . . . Her husband's greatness, and Oliverian power, with some rash expressions that he uttered following too unhandsomely from his mouth, dismayed my daughter. She could not be quieted until I crept privately again the second time into the Chamber, and then I found her words true. I willed her to bring down a foot the which she soon did. But being much disquited with feare of ensuing danger, shee prayed me to carry on the rest of the work."

This last sentence is significant. On the average the female midwife would lack fortitude and determination in times of physical stress to a greater degree than a man. This alone was one reason why the man-midwife steadily extended his practice. It would seem that this was definitely so in the case of Willughby.

Willughby was a most strenuous advocate of version and extraction by the feet (the *handy* operation in his own words) as the best method of overcoming all difficulties in child-birth. His vade-mecum for midwives is indeed entirely devoted to descriptions of this operation—repeated again and again in detail —as the different causes of difficult labour are being considered.

Having so severely criticized the early English writers of hand-books for midwives, it is only fair to record that the great William Harvey himself had written a chapter in his *De Generatione Animalium* on the physiology of labour. He advocated the study and imitation of Nature's processes : "Nature must be our adviser, the path she chalks must be our walk."

Percival Willughby, a friend and great admirer of William Harvey, says: "I know none but Dr. Harvey's directions and methods, the which I wish all Midwives to observe and follow and oft to read over and over again; and in so doing they will better observe, understand, and remember the sayings and doings of that most worthy, good and learned Doctor, whose memory ought to be had forever in great esteem with midwives and child-bearing women." Unfortunately, Harvey's short chapter on normal labour was buried in a learned discourse, and so was inaccessible to the illiterate midwives.

Willughby himself gave sound advice in simple English. He writes: "The midwife's duty in a natural birth is no more but to attend and wait on Nature. . . . Let them always remember that gentle proceedings (with moderate warm keeping and having their endeavours dulcified with sweet words) will best ease and soonest deliver their labouring women." His manuscript can have been accessible to very few; it was not printed and published until 1863, nearly two hundred years after it was written. I possess a manuscript copy of the second portion, *The Countrywoman's Vade mecum*, dated 1741, under the signature of Robert Gell, Surgeon, of Wirksworth, a village only a few miles from Derby, where Willughby had practised.

However, a "popular press" existed even in those days, and the vacuous writings of Culpeper and his like were those most readily accessible to the midwife. You will thus see that English midwives were in great need of the practical text-books written by the really experienced French midwives and accoucheurs which were translated for English readers in the last quarter of the seventeenth and first half of the eighteenth century.

The earliest important work of this epoch was written by François Mauriceau, and translated into English in 1672 by Hugh Chamberlen, a descendant of the Dr. Peter Chamberlen who invented the midwifery forceps, which were still being kept a family secret. Hugh Chamberlen in 1670 went to Paris in the hope of selling the secret. With this object he attempted to deliver a patient of Mauriceau's, a rickety dwarf, but failed miserably, causing a fatal rupture of the uterus. He quickly left France, but a few years later he assisted the progress of midwifery in England by translating the text-book of Mauriceau, who was at the time the greatest living accoucheur. In the preface to this translation Chamberlen criticizes the common method of delivery by means of hooks fastened in the head of the child.

After the publication of this translation Hugh Chamberlen acquired a large practice and made a great fortune. But he and his forebears will never be forgiven for holding up, for their personal gain, knowledge which was so greatly required by suffering women. Not many years later the secret was revealed, and the ability to use forceps was one of the chief reasons for the introduction of more and more men-midwives into the lying-in chamber.

Another practical and useful book was translated into English in 1719. This was *A General Treatise of Midwifery*, by Monsieur Pierre Dionis, Sworn Master Surgeon of Paris. Dionis devotes several chapters to the question, much discussed at the time, whether a woman should be attended by a midwife or a man-midwife. It makes good reading, but I have only time to tell you of his retort to the statement that as *midwives only* are mentioned in the Bible men could not have taken any part in those days. " But,"

says Dionis, "'tis evident that if Eve had any Help in bringing forth her first Children till some of 'em grew up, as 'tis probable she had, she must have been obliged to Adam for it; and that therefore he was the first who did the Office of a Midwife, and it is to be presumed taught the Women the Art of it, so far as he understood it."

Dionis also lays down the necessary attributes of the midwife and of the surgeon who practises midwifery. Amongst these he considers "Midwives ought not only to have all the good Qualities required in Men-midwives, but must also leave off several Vices proper to their Sex and Profession. They are commonly prating Gossips, and fancy that they shall be thought more skilful and able in their way if they tell a thousand wonderful Stories. . . . A Midwife ought to be a Woman of strict Virtue, and extremely tender of her own Character: Her Person ought to be agreeable, her Words few; and she must by no means allow herself to tell wanton Stories, to use Puns, or smutty *double Entendres*, lest she offend against the Modesty of Ladies, and others, to whom she is call'd." In laying down what is required in a surgeon who practises midwifery, he states that "Surgeons ought to be well-bred Men, and skilful and able in their Profession; but especially those who practise Midwifery. Clownishness is somewhat pardonable in an Army, Town, or Hospital-Surgeon; but 'tis intolerable in one who has to do with Ladies, who value themselves upon being more nice than Men, and who are apt to be affronted, if he commits the smallest Blunder, or drops but one unguarded Expression. . . . He must make no Remarks upon what passes in time of Labour; and in a word, he must shew himself a perfect honest Man, who squares all his Actions by the Word of God. He must therefore be virtuous, of a sweet Temper, affable, full of Compassion, and always contented with any handsome or moderate Fee that is given him."

Mauriceau also describes the attributes desirable in an accoucheur. Amongst these "He ought to have a pleasant Countenance and to be as neat in his Clothes as in his Person." But he adds: "Some are of Opinion, that a Practitioner of this Art ought on the contrary to be slovenly, at least very careless, Wearing a great Beard, to prevent the Occasion of the Husband's Jealousy that sends for him."

I have previously quoted from another French author, de la Motte, whose *General Treatise of Midwifery* was translated into English in 1746 at the instigation of William Smellie, our greatest writer on midwifery. Smellie to a considerable extent modelled his own style on that of de la Motte, by giving graphic descriptions of actual clinical cases.

By this time a few English writers had published really useful books based on their own experience.

In 1725 John Maubray published *Midwifery brought to perfection by manual operation.* "An English book is due," he said, "for what books of Midwifery have we had in England but bare Translations?" He also pleaded for the building of a public lying-in hospital in London, pointing out that "we have hospitals for all sorts of indigent people; only on this point of provision for poor miserable women in the time of their natural affliction . . . we have been hitherto and are still deficient, notwithstanding the excellent good precedents set before our eyes in foreign countries." Maubray started the first English school of midwifery. Lectures were read twice a week, but he laid much more stress on the practical tuition.

He advertised that he had "at great expense and trouble provided a sufficient number of pregnant women upon whom the students would occasionally . . . practise the touch; (that is, vaginal examination); and when the women fell in labour the students would have the performance of the deliveries, every one in his turn. At this rate," he boasts, "I flatter myself that in time I may contribute to the stocking of not only London but all Great Britain with a set of as good and expert practitioners of Midwifery as any other country whatsoever may have to boast of." He was entirely opposed to the use of any "chirurgical tools," except "that only which Nature designed, the hand."

In 1733 Edmund Chapman, in *An Essay on the Improvement of Midwifery*, gave the first description (and in the second edition, in 1735, a drawing) of the midwifery forceps. He writes: "As to the Forceps, which, I think, no Person has yet any more than barely mentioned, it is a noble instrument, to which many now living owe their Lives, as I can assert from my own knowledge and long successful Practice." Chapman had started a school in London in which "to instruct young gentlemen in the art of midwifery."

In 1734 William Giffard's *Cases in Midwifery* appeared. This book contains the earliest recorded description of the use of midwifery forceps on an actual case, delivered on 20th April, 1726.

In 1739 Sir Richard Manningham, a fashionable physician, opened a lying-in hospital in Jermyn Street, Westminster; this was the first General Lying-in Hospital in the British dominion; it was the actual forerunner of Queen Charlotte's Hospital. Manningham also had a school for young physicians, surgeons and women. He advertised that he taught

"the performance of deliveries of all kinds, even the most difficult, with the utmost decency and dexterity by means of a contrivance made on the skeleton of a woman with an artificial matrix, whereby all the inconvenience which might otherwise happen to woman from pupils' practising too early on real objects will be prevented; for by this method and contrivance each pupil will become in a great measure a proficient in his business before he attempts a real delivery." Both these ideas, the use of a manikin and the actual delivery of indigent women, *on the district* as we now say, were cribbed from the great French school in Paris.

At last English midwifery practice was beginning to emerge from its deplorable plight. It seems clear that the entry of men into the practice of midwifery had brought about this great change, more especially by the opportunity it gave them for the study of normal labour. The ball had been set rolling, and in a short time there appeared an individual who increased the rate of progression to an amazing degree. This was William Smellie, who has been most justly described as "The Master of British Midwifery." As a matter of fact, there can be no doubt that no other man ever advanced knowledge of the theory and practice of midwifery to an extent in any way comparable with William Smellie.

It is most likely that he was trained by an apprenticeship in Lanark or Glasgow. He commenced practice as an "unqualified practitioner" in Lanark in 1720, when twenty-three years of age. There he practised for nearly twenty years. He tells us that during this time he "was seldom called to deliver women except in laborious and preternatural cases." In 1738 he left Lanark and travelled to London, in order, he writes, to acquire further information on the use of forceps, of which he had read in the books of Chapman (1733) and Giffard (1734).

In 1739 he set up in London as an apothecary and accoucheur, and by 1741 he was established as a teacher of midwifery. His method was largely practical teaching in the slums at the bedside of women whom he supported financially during their lying-in, on condition that he might be allowed to bring his pupils to watch and even take part in the delivery. Systematic lectures were given as well, and operations practised with the aid of a phantom which he had made and which was a great improvement on those used in other schools.

In 1752 he published his *Treatise of Midwifery*, in 1754 a *Collection of Cases* and also a *Sett of Anatomical Plates to illustrate the Treatise.* Finally, during his retirement he finished the record of his life's work by preparing a third volume on *Preternatural Cases in Midwifery.* This was published posthumously in 1764. As the result of the late Professor John Glaister's researches, we know that Tobias Smollett the novelist edited all three volumes of his writings.

He had kept records from the early days of practice, and he must have studied them again and again, adding comments and new observations as time went on, and as he himself says, "losing no opportunities of acquiring improvement in knowledge and cheerfully renouncing those errors which he had imbibed in his early days." The part he took in the development of the midwifery forceps is well known. He laid down rules for their use and warnings against their premature use which have never been improved upon, and are probably more needed in these days than in his own.

Mrs. Dorothy George in her recent study of *London Life in the Eighteenth Century* expresses the opinion that the impulse Smellie gave to midwifery helped to establish a number of lying-in hospitals in London. He was not on the staff of any of the two or three lying-in hospitals which were established in his time. His practice was, in modern phraseology, domiciliary, and he must have often worked under the greatest conceivable difficulties, overcoming them by gentleness, skill and dogged perseverance. Difficult versions in cases of impacted shoulder presentations, without of course the help of anæsthesia, are described in detail. On one occasion he writes: "This case so fatigued me, that I was obliged to shift, and go to bed after I was carried home in a chair. My hands were so swelled that I could only use them like a gouty person for a day or two." (Case 384 in McClintock's edition.)

He was the greatest success possible, and crowds of men, largely from the army and navy, attended the classes. Unfortunately, though not unnaturally, much jealousy was aroused, and pamphlets and even books were printed criticizing and condemning his methods: caricatures and lampoons characteristic of the period were issued. One critic said he "was uncouth in appearance and rough in manner, with large hands fit to stretch boots in Cranburne alley," a jibe which I fear is lost on us. A Dr. Douglas accused him of having outside his house a paper lantern inscribed with the words: "Midwifery taught for five shillings." However, the chief controversy was the old one of man versus woman. In 1760 a certain Mrs. Nihell, who has been described as "a rough-tongued jealous midwife," published a book of over 400 pages in which she reviled men-midwives in general and William Smellie in particular.

She was trenchant and remorseless in her criticisms. These were reasonable enough when levelled against the brutal measures employed by not a few ignorant men, who used crotchets, hooks and other mutilating instruments to overcome simple causes of delay which a good midwife like Mrs. Nihell herself, and a sound, though self-taught, obstetrician like Smellie, knew could be relieved by suitable soporific drugs and patience. She reviled some writers of treatises on midwifery for vainly pretending "to the triple union of the characters of man-midwife, surgeon and physician in one person," and "it will be found," she says, "that all their boasted superiority of erudition, has only led them into the greater errors of practice, and the most barbarous violences to nature." We cannot afford time to-night to enter into the squabble, it is a big subject in itself.

Smellie was a reserved man: though publicly attacked he did not retaliate. As a matter of fact, he did more than anyone else to overcome prejudice against the use of midwifery forceps and against male practitioners. His reputation for judgment, candour and fairness led to him being called in to settle disputes between doctors and midwives.

During and immediately after his time the number of men-midwives increased rapidly. In the oldest Sheffield directories for 1797 and 1798 fourteen citizens were given the title of surgeon *and man-midwife* in contrast to a number labelled physician. The title was not appreciated, it would seem, and by 1828 the designation had disappeared from the directory, in Sheffield at any rate.

Although Smellie did not publicly enter into this dispute, he made it clear in his treatise of 1752 that he believed there was room and necessity for the man accoucheur as well as the woman midwife. In his description of the requisite qualifications for accoucheurs and for midwives he pleads, in his kindly way, for the friendly co-operation of the two. This attitude of mind gradually spread and the rivalries abated, to flare up from time to time during the following fifty years or so. Even as late as 1830 a society was founded, with a certain Sir Anthony Carlisle as the president, for the avowed purpose of regaining for female midwives the position which had been usurped by the man. Sir Anthony even succeeded in getting an address on the subject published in *The Times* of 11th May, 1830.

But the benefits conferred by the male obstetrician of the day had already brought conviction as to his worth, and from then until now a succession of men have practised midwifery sufficiently well to earn the approval and patronage of the general public.

NOTE.—Professor Miles Phillips's paper was originally entitled "Men Midwives Past and Future." The second part, dealing with future developments in obstetrics, will be published elsewhere. In it the value was emphasized of institutional as against domiciliary confinement, and the importance of measures to prevent post-parturition disability in both mother and infant, high among which the author placed central episiotomy.

